# Graph analysis of cortical reorganization after virtual reality-based rehabilitation following stroke: a pilot randomized study

**DOI:** 10.3389/fneur.2023.1241639

**Published:** 2023-10-06

**Authors:** Jamille Almeida Feitosa, Raphael Fernandes Casseb, Alline Camargo, Alexandre Fonseca Brandao, Li Min Li, Gabriela Castellano

**Affiliations:** ^1^Gleb Wataghin Institute of Physics, University of Campinas – UNICAMP, Campinas, Brazil; ^2^Brazilian Institute of Neuroscience and Neurotechnology – BRAINN, Campinas, Brazil; ^3^Neuroimaging Laboratory, Department of Neurology, University of Campinas – UNICAMP, Campinas, Brazil

**Keywords:** stroke, functional connectivity, graph theory, virtual reality, neuroplasticity

## Abstract

**Introduction:**

Stroke is the leading cause of functional disability worldwide. With the increase of the global population, motor rehabilitation of stroke survivors is of ever-increasing importance. In the last decade, virtual reality (VR) technologies for rehabilitation have been extensively studied, to be used instead of or together with conventional treatments such as physiotherapy or occupational therapy. The aim of this work was to evaluate the GestureCollection VR-based rehabilitation tool in terms of the brain changes and clinical outcomes of the patients.

**Methods:**

Two groups of chronic patients underwent a rehabilitation treatment with (experimental) or without (control) complementation with GestureCollection. Functional magnetic resonance imaging exams and clinical assessments were performed before and after the treatment. A functional connectivity graph-based analysis was used to assess differences between the connections and in the network parameters strength and clustering coefficient.

**Results:**

Patients in both groups showed improvement in clinical scales, but there were more increases in functional connectivity in the experimental group than in the control group.

**Discussion:**

The experimental group presented changes in the connections between the frontoparietal and the somatomotor networks, associative cerebellum and basal ganglia, which are regions associated with reward-based motor learning. On the other hand, the control group also had results in the somatomotor network, in its ipsilateral connections with the thalamus and with the motor cerebellum, which are regions more related to a purely mechanical activity. Thus, the use of the GestureCollection system was successfully shown to promote neuroplasticity in several motor-related areas.

## 1. Introduction

According to the World Stroke Organization, “stroke has already reached epidemic proportions,” with 25% of adults over 25 years old estimated to have a stroke in their lifetime (1). Although the proportion of survivors is larger than that of deceased (around 6:4) (1), many of the first remain with physical disabilities. Advances in virtual reality (VR) technologies for motor rehabilitation in the last few years have created new therapy possibilities for stroke victims. With the demand for rehabilitation constantly growing (2), VR systems represent an option that helps to alleviate the scarcity of clinics and physiotherapists, particularly in low-income countries. VR technologies have allowed physical therapy experts to explore new paths to stimulate brain plasticity (3) and improve rehabilitation. Besides, the use of these systems has been related to a higher rate of adherence among patients due to their ludic character (4).

As stated by Weiss and colleagues, VR is the ‘use of interactive simulations created with computer hardware and software to present users with opportunities to engage in environments that appear and feel similar to real-world objects and events’ (5). VR allows the creation of a safe environment where it is possible to perform the daily activities and exercises needed for a motor rehabilitation therapy, while having close supervision of a therapist, be it presential or remote. Several VR systems, both off the shelf and explicitly developed for research, have been used for rehabilitation of upper and/or lower limbs of stroke patients [e.g., (6–9)] and other conditions, such as cerebral palsy (10) and Parkinson’s disease (11, 12). These systems range from non-immersive, where the virtual environment is typically presented on a video display, to fully immersive, where the user usually wears glasses or similar devices that give the impression of being in a different environment (the virtual one) altogether. The first type is more abundant in the literature, possibly because the latter can cause cyber-sickness symptoms (13). Another important aspect in VR systems is the user interface, which can make use of controllers (e.g., joysticks) or be based on gestural control, which can rely on wearable sensors or optical devices. Gesture-controlled VR allows users to perform closer-to-real-life movements, compared to controller-based VR systems. Indeed, gesture-controlled VR systems are known as natural interfaces (14). Although nowadays these systems are equivalent in terms of complexity issues, prices for VR systems with wearable sensors are usually more expensive, and the controllability of a gesture-based VR system is still slightly more difficult than that of a conventional VR system, considering gesture recognition concerns (15).

The GestureCollection system (14) is a low-cost, easy and intuitive setup, non-immersive rehabilitation solution developed within our group, based on the gestural control of the computer. It comprises three VR games: GesturePuzzle, in which users must use the upper limbs to put the pieces of a puzzle together; GestureChess, in which users play a chess game using the upper limbs to move the pieces; and GestureMaps, in which subjects can navigate through the virtual map of Google Street View controlling the virtual movements with real stationary gait and trunk rotation. Here, we investigate brain changes associated with clinical outcomes in stroke patients performing motor rehabilitation with and without the inclusion of GestureCollection-based activities in the therapeutic protocol.

Indeed, a way to investigate the effects of rehabilitation in the brain that has been widely used is resting state (rs) functional magnetic resonance imaging (fMRI) associated with graph theory to measure the topological changes in brain networks (16). fMRI (based on the blood oxygenation level dependent – BOLD – signal) measures a mixture of oxygenation, volume and flow changes in the brain’s blood supply, secondary to its electrophysiological response (17). rs-fMRI has been used to probe concurrent fluctuations in the BOLD signal occurring at distinct brain regions (18), from which have emerged the functional brain networks (19). Brain networks are known to have a small-world topology (20), i.e., they present a high clustering coefficient (CC) and a small average shortest path length. In practice, this implies an optimized balance between the ability to process specialized information (segregation) and to ensure an efficient information flow through the brain (integration). In an injured brain, this balance gets compromised, and it is expected to change as a neuroplasticity effect.

Other papers have reported using fMRI to investigate brain changes resulting from the use of VR solutions for rehabilitation of stroke patients (21–36) (for a more complete review on this topic, see (3)). However, most works used task-based fMRI (22, 24, 26, 29–36), while only a few used rs-fMRI (21, 23, 25, 27, 28). From the latter, only the work by Feitosa et al. actually investigated brain network topology changes using graph theory (23). Therefore, there is room for more research in the topic of network topology changes resulting from VR rehabilitation in stroke patients.

The aim of this work was to investigate changes in functional connectivity and network parameters in stroke victims as a result of motor rehabilitation, performed with or without complementation with GestureCollection. We hypothesize that patients who undergo therapy along with this VR approach are going to show larger increased connectivity with motor-related areas than patients who undergo conventional therapy.

## 2. Materials and methods

### 2.1. Subjects

Fifteen patients with ischemic stroke diagnosis were recruited through the Neurovascular Outpatient Clinic in the Clinics Hospital of University of Campinas and by spontaneous search of volunteers. The patients were assessed for eligibility by a blinded assistant and allocated into groups through alternation. One patient was lost to follow-up and four others had their data discarded during the preprocessing of the MRI data, resulting in a sample of 10 individuals (Table 1).

**Table 1:** Demographics of the final set of patients included in the study.

Subject	Age	Gender	Group	Chronicity (months)	Lesion		Total FMA
					Side	Site	
S1	55	M	Exp	6	L	Caudate	215
S2	50	F	Exp	9	R	Temporal lobe	116
S3	52	F	Exp	10	R	Putamen	204
S4	62	M	Exp	6	R	Microinfarcts	126
S5	62	M	Exp	12	R	Microinfarcts	171
S6	49	M	Con	16	L	Hippocampus	216
S7	66	F	Con	12	R	Putamen	220
S8	68	F	Con	8	R	Fusiform gyrus	210
S9	61	M	Con	8	L	Supramarginal gyrus	204
S10	68	M	Con	8	L	Supramarginal/Postcentral gyrus	221

Exp, experimental group; Con, control group; F, female; M, male; L, left; R, Right; FMA, Fugl-Meyer Assessment.

Inclusion criteria were: (1) patients with ischemic stroke between 6 and 24 months after onset; (2) age 45–70 years; (3) both genders; (4) motor and functional impairment measured by modified Rankin Scale (mRS 1–4); and (5) signed Informed Consent Form. Exclusion criteria were: (1) contraindications to the MRI exam (such as the presence of metallic implants, claustrophobia or other conditions); (2) lack of capacity to understand or follow verbal commands; and (3) serious visual impairments. All research was performed in accordance with the ethical standards of the Declaration of Helsinki and its later amendments. The study was approved by the Ethics Committee of University of Campinas (protocol 60860616.0.0000.5404) and all subjects signed an informed consent form prior to entering the study.

### 2.2. Rehabilitation protocols

Participants were alternated into two groups: the control group received 1 h of “conventional physiotherapy” (CPT) (therapeutic exercises and functional exercises for upper limb and gait), plus 30 min of visual stimulation (video showing movements of lower and upper limbs); and the experimental group received 1 h of CPT followed by the application of VR for 30 min. Rehabilitation sessions were conducted twice a week, for six consecutive weeks (total of 12 sessions). CPT included: joint mobilization, stretching for upper and lower limb and trunk, proprioceptive neuromuscular facilitation exercises, training of postural transfers (sitting, lying and standing) and gait training in a proprioceptive circuit. Clinical data such as heart and respiratory rate, blood pressure and effort reported by the patient were considered for the conduction of the treatment.

### 2.3. VR system

**Figure 1:**
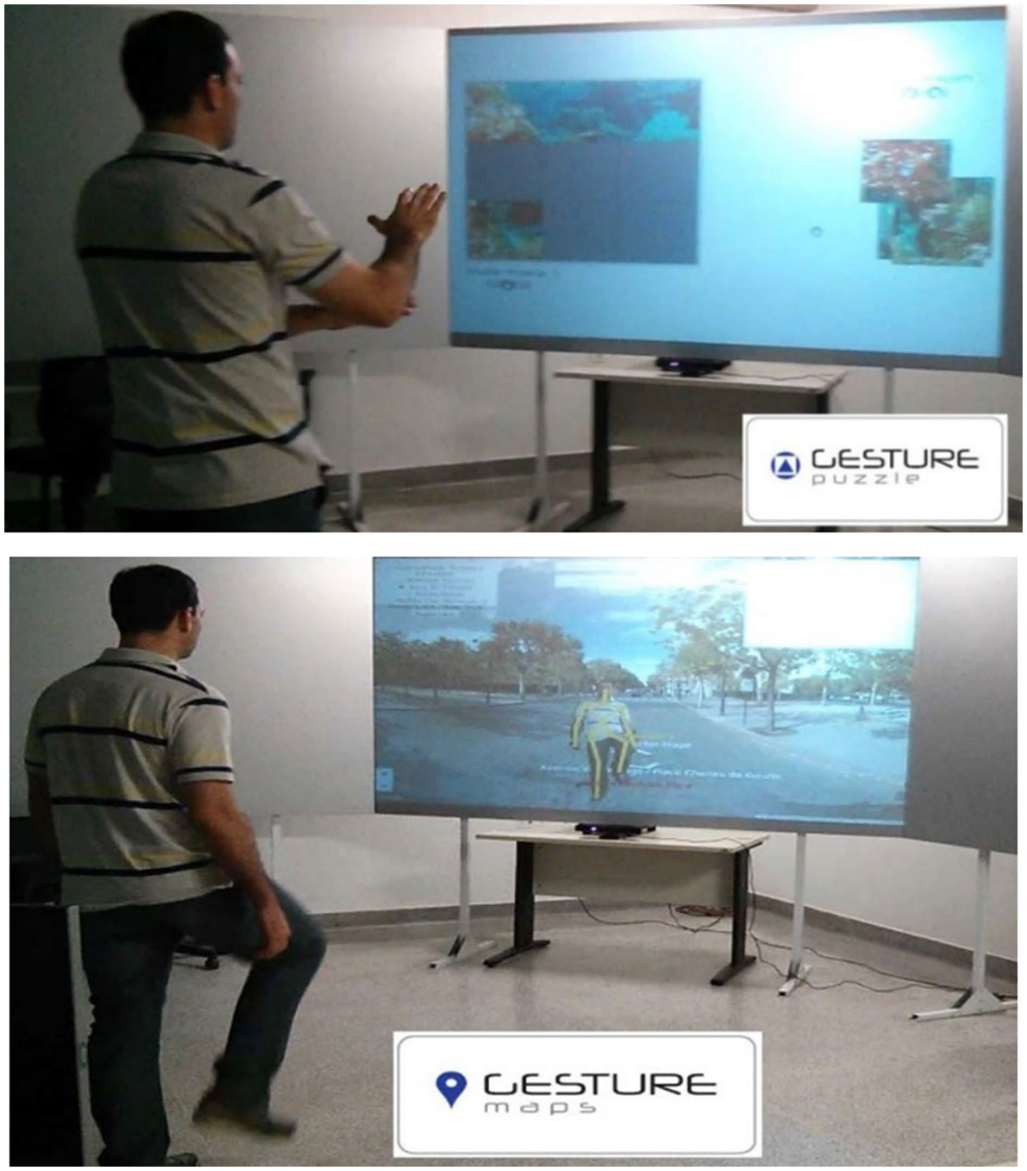
GestureCollection games: Gesture puzzle (top) and Gesture maps (bottom). Figure reprinted with permission from Brandao et al. (14).

**Figure 2:**
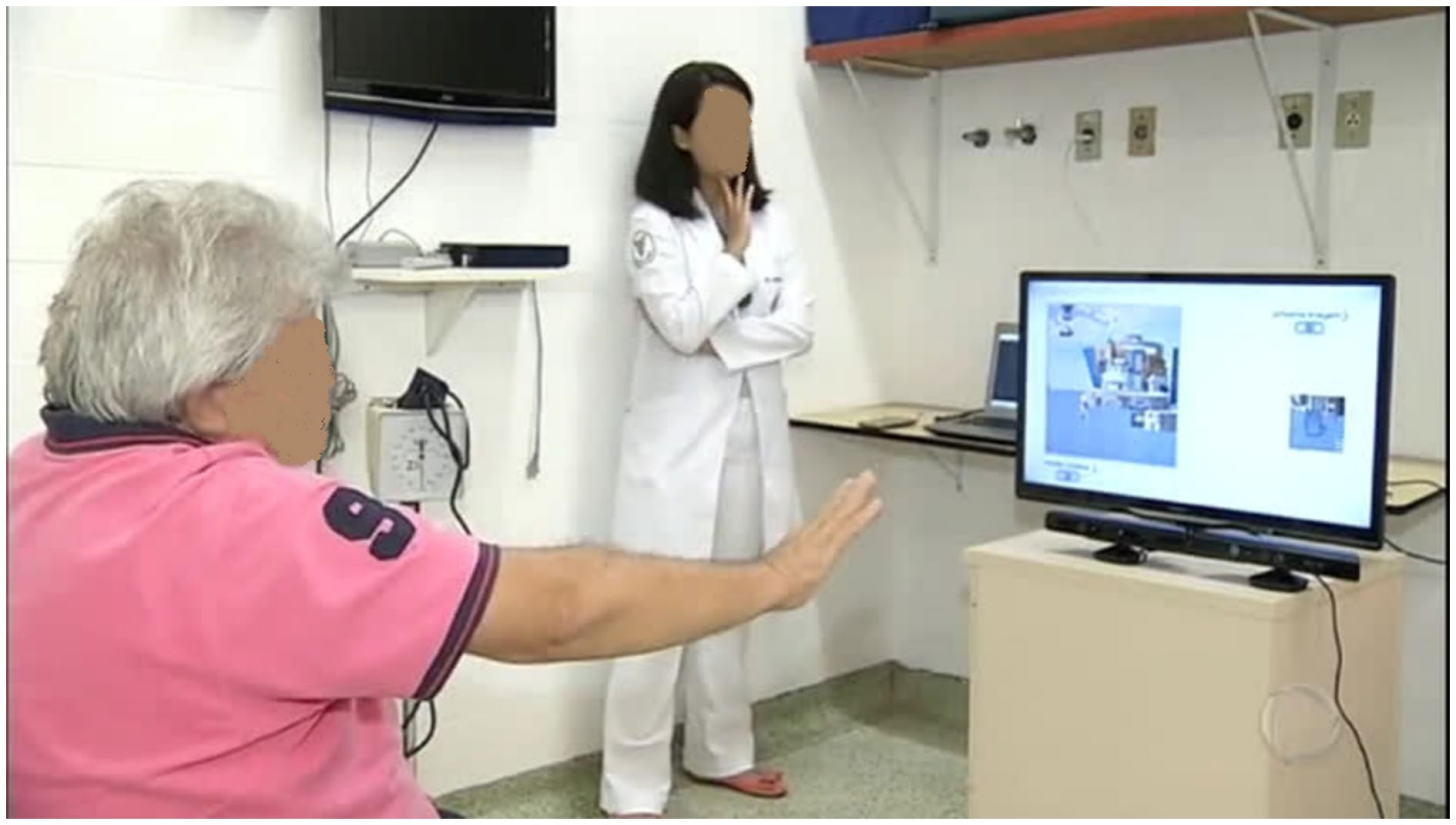
Experimental setup: the patient plays GesturePuzzle seated in front to the screen which reproduces the game. Below the screen, a Kinect captures the user movements.

The GestureCollection VR set (14) was used in the therapy sessions. GestureCollection requires a computer, a monitor and the Kinect 360 gesture recognition sensor (Microsoft, Redmond, Washington). Two games (Figures 1, 2) were selected: (1) Gesture Puzzle, a 9-piece virtual puzzle that must be put together through the shoulder movements of abduction, adduction and flexion, elbow extension, and wrist extension (when possible), with the patient sitting on a chair with no movement restriction of the trunk and upper limbs (Figure 2); and (2) GestureMaps, which consists of navigating through different scenarios of a virtual map, based on gestures of stationary gait – mainly hip and knee flexion, with trunk rotation toward the side the user wants to explore in the map –performed between parallel bars for patient safety.

### 2.4. Clinical evaluations

To verify functional outcomes, we used the Fugl-Meyer Assessment (FMA), the Berg Balance Scale (BBS), the Timed Up and Go (TUG) test and the Montreal Cognitive Assessment (MoCA). Evaluations were conducted at the beginning and the end of the study period by a physiotherapist blind to group assignment.

FMA is considered one of the most complete measures for post-stroke functions (37), consisting of five domains: motor function, sensory function, balance, passive range of motion and joint pain, for the upper and lower limbs. The maximum score is 226 points, 126 points for upper limbs, 86 points for lower limbs and 14 points for balance. The higher the score, the better the functions of the patient.

BBS was used to assess balance (38), consisting of 14 activities, such as sitting or standing up without support, with a total score of 56 points. The higher the score, the better the balance function – with scores below 40 suggesting a risk of falling.

TUG (39) is a test of mobility, gait and balance performed by the following steps while being timed: stand up from a chair, walk forward 3 meters, walk back to the chair, and sit down. The test is conducted three times, and the average time is used.

MoCA (40) is a fast, practical and effective method for cognitive assessment and screening, which evaluates attention, concentration, executive function, memory, language and other functions related to cognition, adding up to 30 points (1 point is mandatory if the patient has 12 years or less of education). A score of 26 or above is considered normal.

### 2.5. Data acquisition and preprocessing

Two MRI acquisitions were performed in a 3.0 Tesla scanner (Achieva, Philips, The Netherlands), one before the first therapy session, and one after the last therapy session. These consisted of a T1-weighted anatomical image (isotropic voxel of 1 mm^3^, field of view (FOV) = 240 × 240 × 180 mm^3^ repetition time (TR) = 6.9 ms, echo time (TE) = 3.2 ms) and T2*-weighted functional images (voxel size = 3 × 3 × 3 mm^3^, no gap, FOV = 240 × 240 × 117 mm^3^, TR = 2 s TE = 30 ms, flip angle = 90, 180 volumes).

Data were preprocessed in Matlab (MathWorks, version R2017b). First, the reorientation of the images to the anterior commissure was manually performed in the SPM12 toolbox. Further steps of the preprocessing were performed in the software UF^2^C (41), which runs in Matlab: (i) functional images were motion corrected by a six-degree-of-freedom rigid body transformation (x, y and z translations and pitch, yaw and roll rotations); (ii) functional images were coregistered to the structural image; (iii) the structural image was segmented into gray matter (GM), white matter (WM) and cerebrospinal fluid (CSF); (iv) functional and structural images were normalized to the MNI template; (v) functional images were spatially smoothed; (vi) the mean WM and CSF signals were regressed out from the functional images; (vii) functional images were temporally filtered to frequencies between 0.08 and 0.1; and (viii) motion parameters estimated in (i) were used as an image quality estimator. Subjects that had exceeded a 0.5 mm (of motion) threshold in more than 70 volumes were excluded.

The original dataset was composed of two groups of seven subjects. Four subjects (two from each group) were excluded due to excessive movement during fMRI, resulting in five subjects per group (Table 1). To perform the group analyses, images from subjects who had lesions in the left hemisphere were flipped so that all lesions would be on the same side (right hemisphere).

#### 2.5.1. Parcellation of the brain

Regions of interest (ROIs) were derived from the MIST functional atlas (42) containing 122 brain divisions already categorized into networks. We split ROIs into left and right hemispheres and created larger ROIs (by combining smaller ones) to have a more general representation of each network. We ended up with a total of 16 ROIs divided into the following five networks: somatomotor, frontoparietal, cerebellum, and basal ganglia & thalamus. These ROIs are summarized in Supplementary Table S2, while the original ROIs and their combination to give rise to these 16 are shown in Supplementary Table S1.

Time series extraction was conducted as follows: each ROI had its mask applied to the functional image. Null time series, relative to borders or voids, and time series whose correlation coefficient to the mean were smaller than two standard deviations were excluded. Finally, the mean was computed again with the remaining time series.

A 16 × 16 adjacency matrix was built for each subject using the Pearson correlation values of each pair of ROIs.

### 2.6. MRI data analysis

#### 2.6.1. Functional connectivity analysis

##### 2.6.1.1. Weighted difference matrices

In this analysis, we sought the pairs of regions with the highest connectivity difference between the pre- and post-MRI. To this end, the negative correlations were excluded from the weighted adjacency matrix (wAM) of every subject and, for each subject, the difference matrix (wdM) of the positive correlations between the timepoints was computed, through the subtraction of pre from post wAM:


(1) $$wdM=wAM_{\mathrm{post}}-wAM_{pre}$$

A *t*-test was applied to each wdM, generating *t*-value difference matrices (t_wdM). The t-values were calculated as follows:


(2) $$t=\frac{r\sqrt{n-2}}{\sqrt{1-r^{2}}}$$

where r was the value of the correlation difference in each connection.

The resultant group weighted difference matrix (gwdM) was obtained through the median of the t_wdM matrices from the group’s subjects:


(3) $$\mathrm{gwdM}=\mathrm{median}\left(t:wdM\left(subj_{1}:subj_{n}\right)\right)$$

where n is the number of subjects.

The *p*-values were calculated for the gwdM matrix and filtered for significance ( p<0.01 ). The Holm-Bonferroni correction for multiple comparisons was applied to the uncorrected significant data leading to no significant results. Therefore, we present here for discussion the uncorrected results with a confidence interval of 99%. The gwdM of each group was plotted as a heatmap. For the matrices’ elements above the diagonal, only significant values were plotted.

##### 2.6.1.2. Changes in network topology

Out of all the established graph metrics, we used the strength (S) and the clustering coefficient (CC) to measure changes on the network topology (43).

To mathematically describe the measures, we must consider some definitions. In a weighted network formed by the set N of n nodes, the indices i and j represent different nodes contained in N ; $a_{ij}$ assumes the value 1 (one) if the nodes i and j are connected or 0 (zero) otherwise; $w_{ij}$ is the weight of this connection, it is a value in the interval $\left[0,1\right]$ ; and $K_{i}$ is the number of connections the node i has.

*S* is the most basic measure in a weighted network. It represents the sum of the weights from the connections that a node has:


(4) $$S_{i}=\overset{n}{\underset{j=1}{\sum }}w_{ij}$$

CC measures how much the neighborhood of a node is connected to itself. It is quantified by the number of triangles formed by the nodes directly connected to the node of interest. A densely connected network region is associated with specialized local information processing. The expression for CC is:


(5) $$CC_{i}^{w}=\frac{1}{S_{i}\left(K_{i}-1\right)}\underset{jh}{\sum }\frac{w_{ij}+w_{ih}}{2}\;a_{ij}a_{ih}a_{jh}$$

Relative differences in network parameters, from the first to the last scans, were computed for *S* and CC. For these computations, only the positive correlations in the wAM were considered. The group result for each study was obtained through the median of the subjects’ relative differences. Considering the generic parameter *P*, the relative difference (or relative mean variation, RMV) was computed as follows:


(6) $$RMV\left(P\right)=\Delta P=\frac{P_{\mathrm{post}}-P_{pre}}{P_{pre}}$$

## 3. Results

### 3.1. Clinical evaluations

The results of the clinical evaluations performed with FMA, BBS, TUG and MoCA for both groups are shown in Table 2. This table shows the individual scores as well as the mean and standard deviation for before and after the intervention, and the RMV [Eq. (6), in percentage].

**Table 2:** Scores of the clinical scales for the experimental (Exp) and control (Con) groups.

Group	Subject	FMA															
		UE – S/E		UE – H/W		LE		Balance		Total		BBS		TUG		MoCA	
		Pre	Post	Pre	Post	Pre	Post	Pre	Post	Pre	Post	Pre	Post	Pre	Post	Pre	Post
Exp	S1	10	13	0	0	16	17	7	8	215	216	47	53	19	22	17	24
	S2	21	24	11	7	22	25	9	10	116	132	23	31	135	90	25	23
	S3	40	40	24	23	32	32	11	11	204	212	47	47	18	19	20	19
	S4	8	10	2	4	24	31	8	10	126	160	36	45	39	43	16	18
	S5	42	40	24	24	24	28	12	12	171	174	38	37	27	26	18	23
Mean		24.2	25.4	12.2	11.6	23.6	26.6	9.4	10.2	166	179	38.2	42.6	47.6	40.0	19.2	21.4
Std		16.13	14.3	11.5	11.2	5.7	6	2.1	1.5	45	36	9.9	8.6	49.6	29.5	3.6	2.7
RMV (%)		5		−4.9		12.7		8.5		7		12		*−*16		11	
Con	S6	42	36	24	24	30	33	12	12	216	219	56	56	10	7	24	21
	S7	41	42	24	24	33	34	12	13	220	222	53	55	17	17	18	22
	S8	39	41	24	24	31	33	11	13	210	223	51	56	13	11	23	28
	S9	42	38	21	22	34	34	14	11	204	219	51	56	10	8	16	17
	S10	39	40	22	23	33	33	14	14	221	213	56	53	13	12	20	18
Mean		40.6	39.4	23	23.4	32.3	33.4	12.6	12.6	214	219	53.4	55.2	12.6	11.0	20.2	21.2
Std		1.5	2.4	1.4	0.9	1.6	0.5	1.3	1.1	7	4	2.5	1.3	2.9	4.0	3.3	4.3
RMV (%)		−3		1.7		3.7		0		2		3		*−*13		5	

Std, standard deviation; RMV, relative mean variation [Eq. (6), in percentage]; FMA, Fugl-Meyer Assessment; UE, upper extremity; S/E, shoulder/elbow; H/W, hand/wrist; LE, lower extremity; BBS, Berg Balance Scale; TUG, Timed Up and Go test; MoCA, Montreal Cognitive Assessment.

### 3.2. Weighted difference matrices

**Figure 3:**
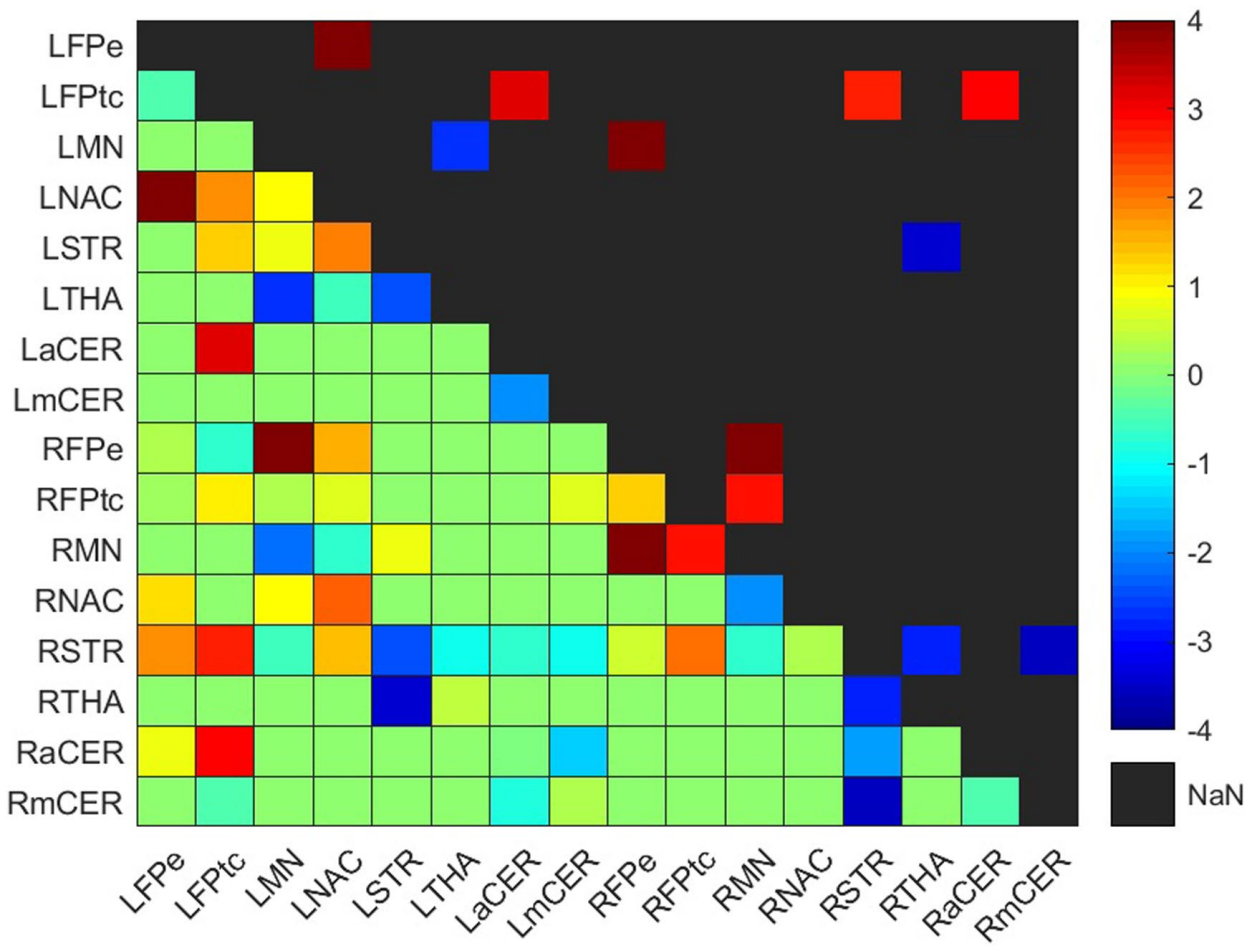
Weighted difference matrix for the experimental group. The lower triangular matrix shows the median *t*-values of the differences between each pair of ROIs after rehabilitation with GestureCollection. The symmetric upper triangle shows only the significant differences (*p* < 0.01). The *t*-values are encoded in the range (−4, 4) in the colorbar.

**Figure 4:**
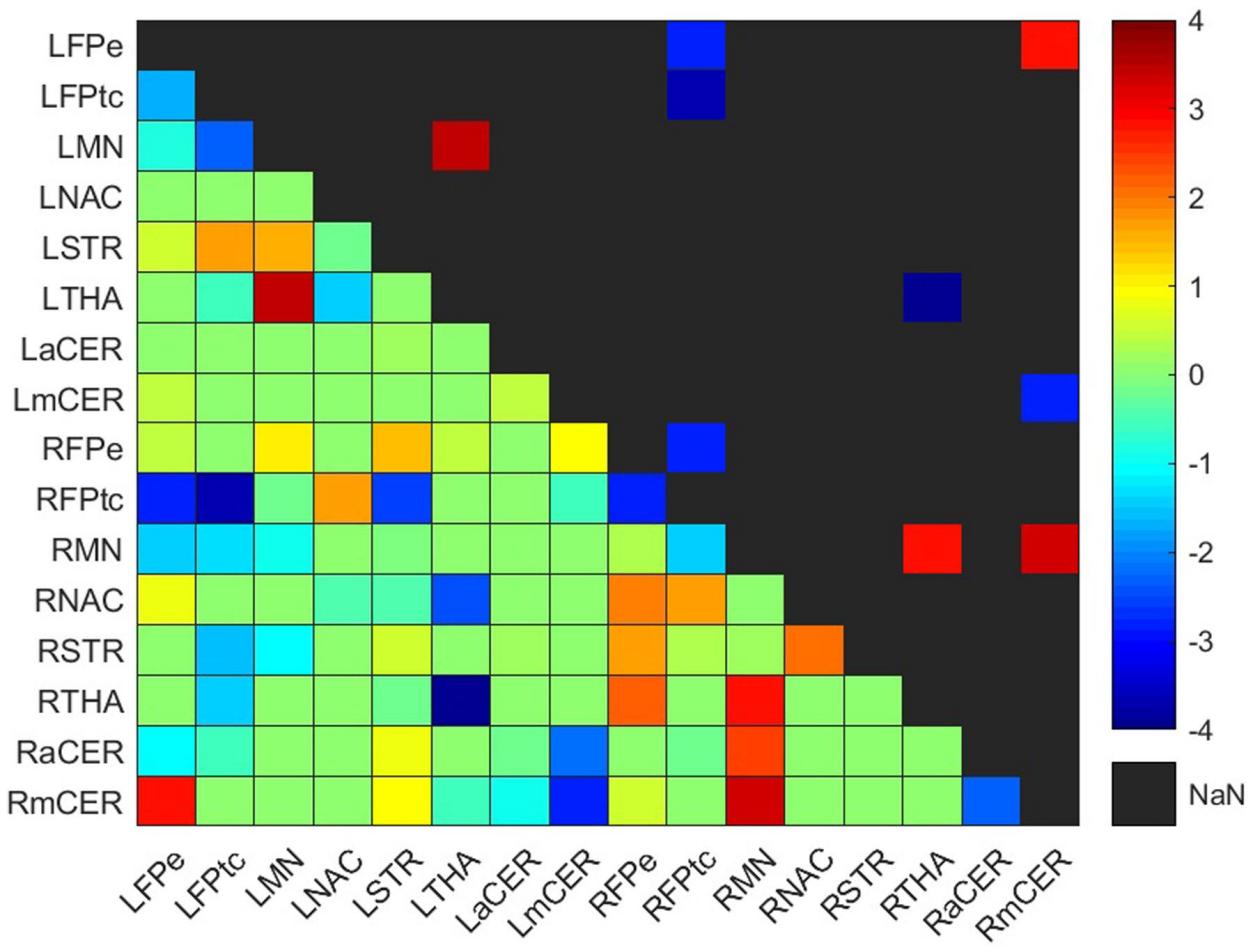
Weighted difference matrix for the control group. The lower triangular matrix shows the median *t*-values of the differences between each pair of ROIs after rehabilitation with GestureCollection. The symmetric upper triangle shows only the significant differences (*p* < 0.01). The *t*-values are encoded in the range (−4, 4) in the colorbar.

Connectivity difference matrices (gwdM) for the experimental and control groups are shown in Figures 3, 4, respectively. Supplementary Table S3 lists the connections that presented significant differences.

#### 3.2.1. Experimental group

In the experimental group, there were seven significant connections with positive differences and four with negative differences (Figure 3; Supplementary Table S3). Two of the increased connections were in the left (contralesional) hemisphere, three were interhemispheric and two were in the right (ipsilesional) hemisphere; the four decreased connections were two in the right hemisphere, one in the left hemisphere and one was interhemispheric.

##### 3.2.1.1. Frontoparietal network

All positive differences involved the frontoparietal network. In the left (contralesional) hemisphere, the correlations between the left frontoparietal executive (LFPe) region and the left nucleus accumbens (LNAC) and between the left frontoparietal task control (LFPtc) and the left associative cerebellum (LaCER) increased from the beginning to the end of the treatment. On the right side, the positive differences occurred in the connections between the right frontoparietal task control region (RFPtc) and the right frontoparietal executive region (RFPe) with the right somatomotor network (RMN). Finally, the increased interhemispheric connections were between the RFPe and the left somatomotor network (LMN) and between the LFPtc and the right associative cerebellum (RaCER) and right striatum (RSTR).

##### 3.2.1.2. Basal Ganglia & Thalamus

Both ipsilesional decreased connections involved the right dorsal striatum (RSTR), in one case with the right thalamus (RTHA) and in another with the right motor cerebellum (RmCER). The interhemispheric decreased connection involved the RTHA and the left dorsal striatum (LSTR). In the contralesional hemisphere, the decreased connection was between the LMN and the left thalamus (LTHA).

#### 3.2.2. Control group

Four increased and five decreased connections were found in the control group difference matrix (Figure 4; Supplementary Table S3). Two positive and one negative differences occurred in the ipsilesional hemisphere, one positive difference was in the contralesional hemisphere, and one positive and four negative differences were interhemispheric.

##### 3.2.2.1. Frontoparietal network

One connection presented a positive difference and three showed negative differences in the frontoparietal network. The positive difference was in the interhemispheric connection LFPe-RmCER. The negative differences were between the right and left frontoparietal network, RFPtc-LFPe and RFPtc-LFPtc, and in the ipsilesional intra-network connection RFPtc-RFPe.

##### 3.2.2.2. Somatomotor network

Three positive differences occurred in the somatomotor network, two in the ipsilesional and one in the contralesional hemisphere. On the right side, the RMN had positive differences with the RTHA and the RmCER; while on the left side, the LMN had an increase in its connection with the LTHA.

##### 3.2.2.3. Basal Ganglia & Thalamus

As mentioned, the thalamus had increased connections with the somatomotor networks at both hemispheres (RTHA-RMN and LTHA-LMN). However, a negative difference occurred in the interhemispheric connection between both thalami, RTHA-LTHA.

##### 3.2.2.4. Cerebellum

The RmCER had two (already cited) positive differences: in the ipsilesional connection RmCER-RMN and in the interhemispheric connection RmCER-LFPe. A negative difference occurred in the interhemispheric connection between the right and left motor cerebellum (LmCER), RmCER-LmCER.

### 3.3. Changes in network topology

**Figure 5:**
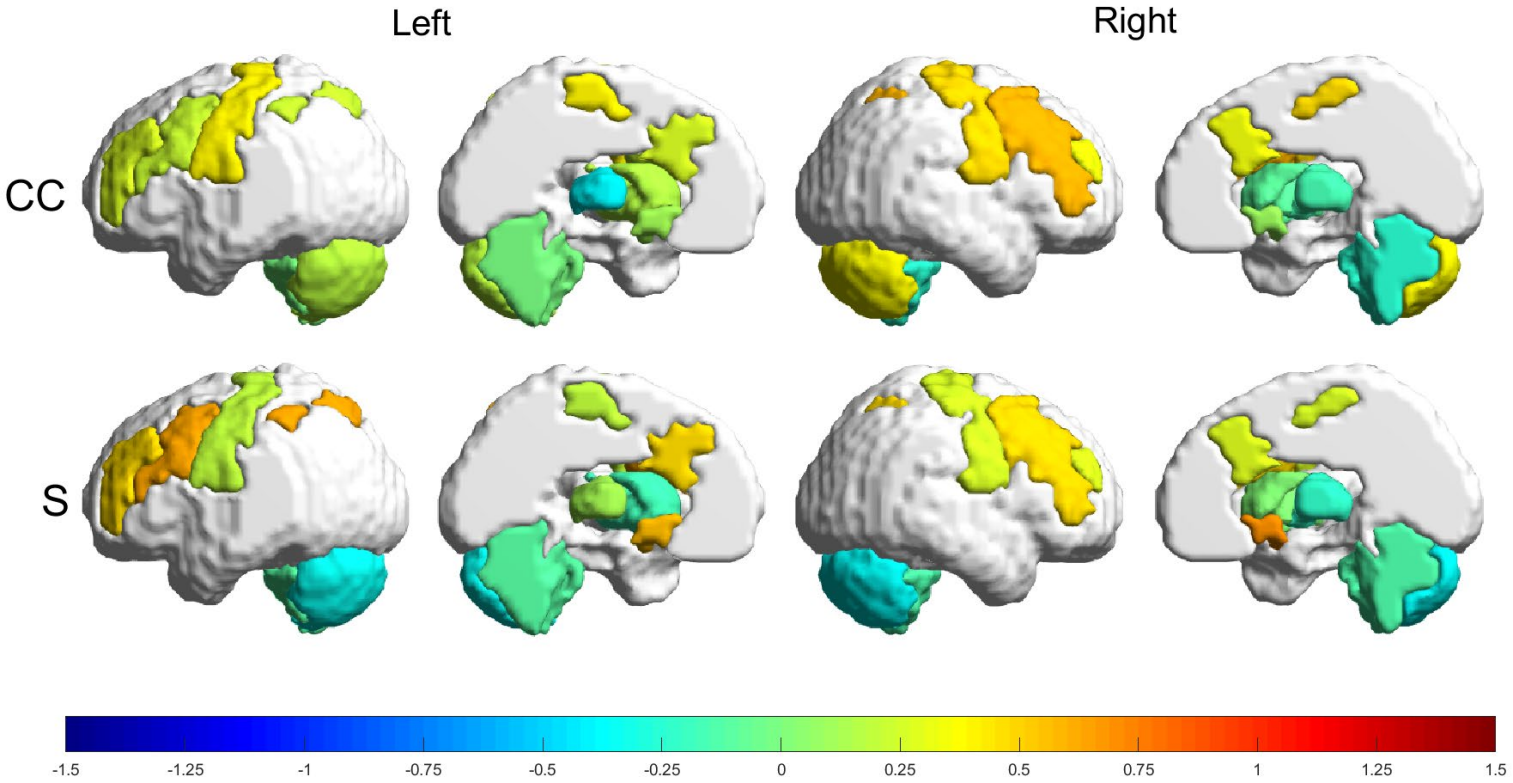
Relative changes [RMV, Eq. (6)] in graph parameters for the experimental group. The intensity of the changes can be seen encoded in the colorbar in the range (−1.5, 1.5). From top to bottom: CC and S. Left to right: left lateral view, left medial view, right lateral view and right medial view.

**Figure 6:**
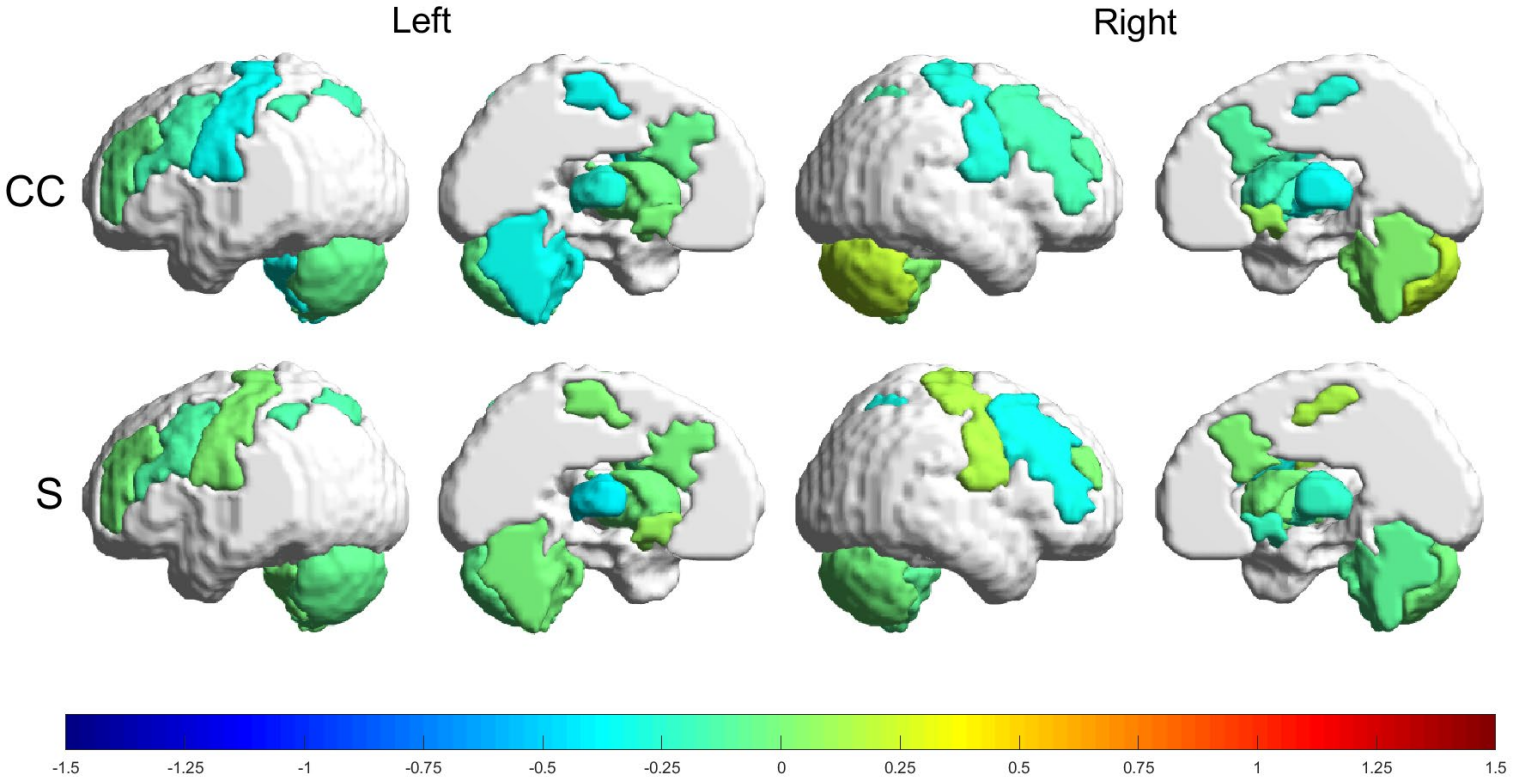
Relative changes [RMV, Eq. (6)] in graph parameters for the control group. The intensity of the changes can be seen encoded in the colorbar in the range (−1.5, 1.5). From top to bottom: CC and S. Left to right: left lateral view, left medial view, right lateral view and right medial view.

Relative changes (RMV) in CC and S [according to Eq. (6) in Section 2.6.1] can be seen as heatmaps plotted on a brain surface (in Figure 5 for the experimental group and in Figure 6 for the control group) and as tabular data (Table 3), where the results were grouped, according to their value, in the ranges: (−0.75, −0.5], (−0.5, −0.25], [0.25, 0.5), [0.5, 0.75) and [0.75, +∞).

**Table 3:** Relative changes [RMV, Eq. (6)] in graph parameters for the experimental and control groups.

Regions	Experimental		Control	
	CC	S	CC	S
LFPe	0.27	0.49	−0.07	−0.01
LFPtc	0.23	0.64	−0.22	−0.17
LMN	0.41	0.19	−0.47	0.03
LNAC	0.12	0.58	0.00	0.12
LSTR	0.17	−0.17	−0.03	−0.03
LTHA	−0.39	0.13	−0.39	−0.54
LaCER	−0.01	−0.11	−0.45	−0.01
LmCER	0.18	−0.35	−0.09	−0.09
RFPe	0.34	0.28	−0.16	0.24
RFPtc	0.60	0.49	−0.21	−0.23
RMN	0.48	0.26	−0.37	0.32
RNAC	0.09	0.70	0.13	−0.06
RSTR	−0.10	0.02	−0.25	0.07
RTHA	−0.16	−0.23	−0.46	−0.19
RaCER	−0.25	−0.14	0.04	0.02
RmCER	0.38	−0.36	0.27	0.03

Changes were highlighted, according to their intensity, in the following ranges: (−0.75, −0.5] (medium blue); (−0.5, −0.25] (light blue); [0.25, 0.5) (light yellow); and [0.5, 0.75) (medium yellow). CC, clustering coefficient; S, strength. R/LMN, Right/Left somatomotor network; R/LaCER, Right/Left associative Cerebellum; R/LmCER, Right/Left motor Cerebellum; R/LTHAL, Right/Left Thalamus; R/LSTR, Right/Left dorsal Striatum; R/LNAC, Right/Left Nucleus Accumbens; R/LFPtc, Right/Left Fronto Parietal task control; R/LFPe, Right/Left Fronto Parietal executive.

#### 3.3.1. Experimental group

The experimental group had more increases than decreases in all metrics, with the greatest increases being in the strength parameter. The following regions had increases in the range (50, 75%): RFPtc, in CC; and LFPtc, LNAC and the right nucleus accumbens (RNAC), in S. Smaller increases, in the range (25, 50%), were found in LFPe, LMN, RFPe, RMN and RmCER, in CC; and LFPe, RFPe, RFPtc and RMN, in S. Decreases in the range (−25, −50%) can be seen in: LTHA and RaCER, in CC; LmCER and RmCER, in S.

#### 3.3.2. Control group

The control group had more decreases than increases in the graph metrics, with only two regions where the parameters increased (within the analyzed ranges). Regions with increases in the range (25, 50%) were: RmCER, in CC; and RMN, in S. Regions with decreases in the range (−25, −50%) were: LMN, LTHA, LaCER, RMN, RSTR and RTHA, in CC. Besides, LTHA had a decrease in the range (−50, −75%) in S.

## 4. Discussion

Clinical scales were used to assess physical and cognitive improvements in the subjects submitted to rehabilitation: FMA, BBS, TUG and MoCA. The improvement in FMA, BBS and MoCA is associated with an increase in these scales, while for TUG improvement is related to a decrease in the corresponding value. The RMV [Eq. (6)] of FMA, BBS and MoCA were larger in the experimental group than in the control group, while the RMV of TUG was smaller for the former group than for the latter (Table 2). However, if we look at the individual subjects, we see that, in general, the control group had more homogeneous values for the scales than the experimental group. Particularly for the TUG scale, we see that S6 in the experimental group dominated the RMV, since he/she had a huge decrease in time, but most other subjects in this group increased their times while only one subject (S9) had a very small decrease. Conversely, in the control group most subjects decreased their time, although in a very discreet fashion, while only one subject (S2) remained with the same time. Nevertheless, most subjects in both groups (with exception of S10 in the control group), improved their total FMA punctuation. Regarding BBS and TUG, 3/5 subjects in both groups increased their punctuation. Therefore, considering all the clinical scales together, subjects showed improvement in their clinical conditions in both groups.

The analysis of group connection changes resulted in more connection increases than decreases for the experimental group (7 vs. 4 for the experimental group), while for the control group, there were fewer connection increases than decreases (4 vs. 5).

Looking more closely at the increased connections (Figures 3, 4; Supplementary Table S3), we see that the experimental group had increases mainly in connections involving the frontoparietal network, the basal ganglia and the cerebellum. Meanwhile, the control group had one increased connection among frontoparietal and cerebellar regions and presented increased connections in the motor network and thalamus. The frontoparietal network is mainly responsible for executive function and attention control (44). Therefore, in this case, we can conjecture that, for stroke subjects, VR training increases attention demand for control of the VR applications. The basal ganglia regulate voluntary movements (45); they are responsible for the inhibition of some motor systems to allow the selection of a given motor action (46, 47). This may imply that VR training calls for more specific actions than conventional training. The cerebellum is responsible for movement coordination and finetuning, motor learning (48) and cognitive functions (49). Thus, increasing connections involving these regions might be associated with improving motor coordination in both VR and conventional training patients. Finally, the thalamus relays signals coming from the basal ganglia and cerebellum to the motor cortex (50). The thalamus is essential in the triggering/initiation of planned movements, where it is activated by a go-cue (51, 52). The increases in thalamus connectivity with the motor cortex, in the control group, can be related to this fact, since in conventional physiotherapy the movement is usually initiated after the command of a second person (the physiotherapist).

Regarding the topology of the networks, both groups presented changes in all analyzed parameters (CC and S) (Table 3). The experimental group had several changes greater than 50%. Conversely, the control group presented one parameter decrease of more than 50%. These changes were for both CC and S in the bilateral nucleus accumbens and frontoparietal task control network for the experimental group and in S in the contralesional thalamus for the control group. Strength (S) measures the sum of the connection weights that reach a given node, and CC measures the amount of local connections around a given node (43). Therefore, we can conjecture that the GestureCollection VR tool had a major influence in increasing and making more efficient the connections related to goal-directed cognition (53, 54) and to reward and reinforcement learning (55). Finally, the control group did not present large increases in any parameter; conversely, it was also the only one to present a large (above 50%) decrease in one graph parameter, namely S in the thalamus. We found no previous results in literature to help justify the decreased connectivity in the thalamus in stroke rehabilitation. Although in Wang et al. (56) the authors also reported decreased connectivity in the thalamus and basal ganglia, they argue that this result can be justified by the subcortical nature of the lesions (common between the subjects in the study).

It is important to highlight that there is a lack of literature on resting state functional connectivity and network changes after motor rehabilitation with VR systems for a direct comparison. Nevertheless, some studies have presented brain activation results in similar areas to the ones found in the present work. Activations in the prefrontal/frontoparietal cortex were found in Orihuela-Espina et al. (26) after VR therapy with stroke patients; in Ossmy et al. (12) after VR therapy in a subject with hemiparkinsonism; and in Ekman et al. (22) after VR training in stroke patients with spatial neglect. Cerebellum recruitment was reported in Orihuela-Espina et al. (26) with stroke patients; and in Maidan et al. (11), with Parkinson’s patients. In more general studies (57), discussed the importance of the correlation between motor-frontoparietal connectivity and motor increased outcomes; and Hordacre et al. (58) showed that improvements in motor performance of stroke patients with corticospinal tract damage are correlated to increased connectivity in the ipsilesional frontoparietal network.

Nevertheless, comparing both treatments underwent by the groups in this study, one main differential of VR rehabilitation is that it is more engaging than conventional treatments. Also, VR allows for the use of several mechanisms to stimulate the user’s brain. The GesturePuzzle application uses false positive feedback, i.e., it amplifies the user movements (in case of need) to aid the user to complete the task, which stimulates reward-based learning mechanisms. In GestureMaps, the user’s body and movements are reproduced in the screen, which can give a sensation of immersiveness and allow action observation, which activates the brain in regions related to motor function. GestureCollection games also stimulated cognition of the participants.

Notwithstanding, the experimental group was composed of younger subjects than those of the control group (mean ages were 56.2 ± 5.6 and 62.4 ± 8.0, respectively). Yoo et al. (59) found that patients with less than 70 years recovered differently from stroke compared to patients over 70 years old - the former group had good functional recovery while the latter had functional decline between 6 to 30 months after stroke. Since our control group had ages closer to this 70-years-old boundary, this might also have influenced the results.

Finally, it is necessary to add that, since we were not able to collect longitudinal data, i.e., to evaluate subjects a few weeks or months after the end of the intervention, we cannot assert that “learning” has effectively occurred, because we do not know if the neuroplasticity changes had a lasting effect. In future studies we intend to perform such a follow up.

The main limitation of this work was the small sample size. This is a recurrent difficulty in this kind of work. More than 50% of the stroke-related studies revised in Feitosa et al. (3) had up to 10 subjects. Besides, some subjects’ data were excluded in the preprocessing step due to excessive movement. Their own health conditions can make it harder for the subjects to stay still during the MRI acquisition.

Another major difficulty was the data’s heterogeneity. With such a small sample, it was not possible to select subjects with more similar lesion locations and clinical conditions, which would be the perfect scenario. Also, the groups were very dissimilar at baseline, with the control group presenting scores close to the maximum on the FMA and BBS scales, and low values at the TUG test, while the experimental group was much more heterogeneous and had worse scores on those scales. This made it difficult to compare the clinical improvement between the two groups. Besides, the subjects with lesions in the left hemisphere had their data flipped with respect to the x-axis, in order to homogenize the lesion side, which can also be an issue, since the brain’s hemispheres are not completely functionally symmetric (60). Other limitations were the size of the screen in which the VR applications were displayed, which was smaller than in laboratory setups (see Figure 1 – laboratory setup – and Figure 2 – experimental setup), the internet connection that wasn’t very good, which in some cases resulted in interruptions in the running of the GestureMaps application. For future studies we are acquiring a larger screen and moving the experimental setup to another location (with better internet).

Finally, in this study only ischemic stroke patients were enrolled, due to this type of stroke being more common and homogeneous. Future studies should also include hemorrhagic stroke patients.

In summary, patients in both experimental (with GestureCollection complementation) and control (without GestureCollection complementation) groups showed improvement in clinical scales after therapy. Despite the difficulty in comparing those clinical outcomes due to the groups’ disparity, in terms of brain changes, we can say that the differences between the groups were unequivocal. VR treatment induced changes in regions that are related to learning, planning and motor execution, while conventional treatment had limited effects in terms of neuroplasticity. There were more increases in functional connectivity in the experimental group than in the control group. The use of the VR system promoted changes mainly in the frontoparietal networks, strengthening the connections between the frontoparietal and motor cortex in the ipsilesional side and interhemisphericaly. The increase in strength in the nucleus accumbens, in both sides, shows how these regions became more important in the network, promoting reward-related motor learning in the subjects that used GestureCollection. Therefore, the use of the GestureCollection system in addition to conventional treatment was successfully shown to promote neuroplasticity in motor-related areas. Nevertheless, the evaluated patient sample was small, and further studies should be conducted to confirm these results. This is the first work of such type performed with stroke patients and the GestureCollection system, and its results may direct the adoption of this low-cost solution in clinics and hospitals across the country.

## Data availability statement

The raw data supporting the conclusions of this article will be made available by the authors, without undue reservation.

## Ethics statement

The studies involving humans were approved by Comitê de Ética em Pesquisa da Universidade Estadual de Campinas (Research Ethics Committee of University of Campinas). The studies were conducted in accordance with the local legislation and institutional requirements. The participants provided their written informed consent to participate in this study.

## Author contributions

GC, AC, AB, and LL: conceptualization. JF, RC, GC, AC, and LL: methodology. AB: software. JF, RC, and GC: validation and formal analysis. JF, RC, GC, and AC: investigation. GC and LL: resources, project administration, and funding acquisition. AC and AB: data curation. JF: writing – original draft preparation. RC, AB, LL, and GC: writing – review and editing. GC, RC, and LL: supervision. All authors have read and agreed to the published version of the manuscript.
