# Supplementary material for: Graph analysis of cortical reorganization after virtual reality-based rehabilitation following stroke: a pilot randomized study
Source: Front Neurol. 2023 Oct 6;14:1241639. doi: 10.3389/fneur.2023.1241639 (PMC10587561; doi:10.3389/fneur.2023.1241639)
Supplement: Supplementary file 1 [file Data_Sheet_1.pdf]

Supplementary information - Graph Analysis of Cortical Reorganization after Virtual Reality-Based Rehabilitation Following Stroke: a pilot randomized study

Table S1. Four networks related to motor function were used in the brain parcellation: Somatomotor Network; Cerebellum; Basal Ganglia; and Fronto-Parietal Network. ROIs listed in the column "Sub-regions" were extracted from the multi-atlas MIST (Urchs et al., 2019) and combined to form 16 bigger ROIs, listed in columns "Label" and "Region".

Network	Label	Region	Sub-regions
Somatomotor network	RMN	Right somatomotor network	Right somatomotor network anteromedial<br>Right somatomotor network medial<br>Right somatomotor network mediolateral<br>Right somatomotor network dorsolateral<br>Right somatomotor network lateral<br>Right somatomotor network ventrolateral
	LMN	Left somatomotor network	Left somatomotor network anteromedial<br>Left somatomotor network medial<br>Left somatomotor network mediolateral<br>Left somatomotor network dorsolateral<br>Left somatomotor network lateral<br>Left somatomotor network ventrolateral
Cerebellum	RaCER	Right associative cerebellum	Right cerebellum VI posterior<br>Right cerebellum VIIab<br>Right cerebellum VI anterior<br>Right cerebellum IX middle<br>Right cerebellum I-V<br>Right cerebellum VI dorsal<br>Right cerebellum IX ventral<br>Right cerebellum IX dorsal
	LaCER	Left associative cerebellum	Left cerebellum VI posterior<br>Left cerebellum VIIab<br>Left cerebellum VI anterior<br>Left cerebellum IX middle<br>Left cerebellum I-V<br>Left cerebellum VI dorsal<br>Left cerebellum IX ventral<br>Left cerebellum IX dorsal
	RmCER	Right motor cerebellum	Right cerebellum VIIb medial<br>Right cerebellum VIIb lateral<br>Right cerebellum crusII anterior<br>Right cerebellum crusI<br>Right cerebellum crusII posterior

Network	Label	Region	Sub-regions
Cerebellum	LmCER	Left motor cerebellum	Left cerebellum VIIb medial<br>Left cerebellum VIIb lateral<br>Left cerebellum crusII anterior<br>Left cerebellum crusI<br>Left cerebellum crusII posterior
Basal Ganglia	RSTR	Right dorsal striatum	Right caudate<br>Right putamen posterior<br>Right putamen anterior<br>Right caudate ventral<br>Right caudate dorsal
	LSTR	Left dorsal striatum	Left caudate<br>Left putamen posterior<br>Left putamen anterior<br>Left caudate ventral<br>Left caudate dorsal
	RNAC	Right nucleus accumbens (ventral striatum)	
	LNAC	Left nucleus accumbens (ventral striatum)	
	RTHA	Right thalamus	Right thalamus dorsal<br>Right thalamus ventral
	LTHA	Left thalamus	Left thalamus dorsal<br>Left thalamus ventral
Fronto-Parietal Network	RFPtc	Right frontoparietal task control	Right intraparietal sulcus<br>Right inferior frontal sulcus<br>Right pars orbitalis<br>Right middle frontal gyrus posterior
	LFPtc	Left frontoparietal task control	Left middle frontal gyrus posterorostral<br>Left middle frontal gyrus posterocaudal<br>Left intraparietal sulcus<br>Left inferior frontal sulcus
	RFPe	Right frontoparietal executive	Right anterior cingulate cortex dorsal<br>Right dorsomedial prefrontal cortex posterior<br>Right middle frontal gyrus anterior
	LFPe	Left frontoparietal executive	Left anterior cingulate cortex dorsal<br>Left dorsomedial prefrontal cortex posterior<br>Left middle frontal gyrus anterior<br>Left right frontal pole lateral

Table S2. ROIs used in the study and their respective labels.

Networks	Label	Region
**Somatomotor network**	RMN	Right somatomotor network
	LMN	Left Somatommotor network
**Cerebellum**	RaCER	Right associative Cerebellum
	RmCER	Right motor Cerebellum
	LaCER	Left associative Cerebellum
	LmCER	Left motor Cerebellum
**Basal Ganglia**	RTHA	Right Thalamus
	RSTR	Right dorsal Striatum
	RNAC	Right Nucleus Accubens
	LTHAL	Left Thalamus
	LSTR	Left dorsal Striatum
	LNAC	Left Nucleus Accubens
**Fronto Parietal Network**	RFPtc	Right Fronto Parietal task control
	RFPe	Right Fronto Parietal executive
	LFPtc	Left Fronto Parietal task control
	LFPe	Left Fronto Parietal executive

Table S3. Connections that had significant differences for the experimental group and the control group. Regions belonging to different networks have been grouped using colors: fronto parietal network – blue; somatomotor network – yellow; basal ganglia – purple; cerebellum – orange.

	**Experimental**			**Control**		
**Positive Variations**	RFPtc	-	RMN	LMN	-	LTHA
	RFPe	-	RMN	RMN	-	RTHA
	RFPe	-	LMN	RMN	-	RmCER
	LFPe	-	LNAC	RmCER	-	LFPe
	LFPtc	-	RaCER			
	LFPtc	-	LaCER			
	LFPtc		RSTR			
**Negative Variations**	RTHA	-	LSTR	RFPtc	-	RFPe
	RTHA	-	RSTR	RFPtc	-	LFPe
	LTHA	-	LMN	RFPtc	-	LFPtc
	RSTR	-	RmCER	RTHA	-	LTHA
				RmCER	-	LmCER

References

Urchs, S., Armoza, J., Moreau, C., Benhajali, Y., St-Aubin, J., Orban, P., & Bellec, P. (2019). MIST: A multi-resolution parcellation of functional brain networks. *MNI Open Research*, *1*, 3. https://doi.org/10.12688/mniopenres.12767.2
